# Chemical Characteristics and Biological Potential of *Prunus laurocerasus* Fruits

**DOI:** 10.3390/life15121847

**Published:** 2025-11-30

**Authors:** Mina Todorova, Nadezhda Petkova, Ivan Ivanov, Yulian Tumbarski, Velichka Yanakieva, Ivelina Vasileva, Yoana Barakova, Emiliya Cherneva, Stoyanka Nikolova

**Affiliations:** 1 Department of Organic Chemistry, Faculty of Chemistry, University of Plovdiv, 4000 Plovdiv, Bulgaria; ioana.barakova@gmail.com; 2 Department of Organic Chemistry and Inorganic Chemistry, University of Food Technologies, 26 Maritsa Blvd., 4002 Plovdiv, Bulgaria; nadezhda_petkova@uft-plovdiv.bg (N.P.); ivanov_ivan.1979@yahoo.com (I.I.); ivelinavas@abv.bg (I.V.); 3 Department of Microbiology, Technological Faculty, University of Food Technologies, 4002 Plovdiv, Bulgaria; tumbarski@abv.bg (Y.T.); yanakieva_vili@abv.bg (V.Y.); 4 Department of Chemistry, Faculty of Pharmacy, Medical University of Sofia, 2 Dunav Str., 1000 Sofia, Bulgaria; cherneva@pharmfac.mu-sofia.bg

**Keywords:** antimicrobial activity, antioxidant activity, carbohydrate composition, chemical characteristics, laurel cherry, total phenolics content, total flavonoids content

## Abstract

Fruits of the *Laurocerasus officinalis* Roem., known as cherry laurel, are found in the Black Sea region. This study examines the phytochemical characterization, antioxidant properties, and antimicrobial potential of cherry laurel’s fruits, variety Novita, cultivated in Southern Bulgaria. The study is significant since it examines the phytochemical profile of this variety’s fruits for the first time. The carbohydrate composition of the fruit was identified. The total polyphenols and flavonoids of five fruit extracts (96% ethanol, 70% ethanol, 50% ethanol, 80% methanol, and water) were determined. The antioxidant potential of these five extracts was evaluated by three methods: DPPH, ABTS, and FRAP. We found that the 96% ethanol extract had the highest content of polyphenols and flavonoids and the highest antioxidant activity values by all three methods. A correlation was established between the content of polyphenols, flavonoids, and antioxidant activity based on the calculated correlation coefficient. The antimicrobial potential of methanolic and aqueous extracts of the fruit of the laurel cherry was evaluated against twenty microorganisms. It was found that the methanolic extracts exhibited moderate to high sensitivity against both Gram-positive and Gram-negative strains, yeast *Saccharomyces cerevisiae*, and five fungi, while water extracts had moderately sensitive activity against *Micrococcus luteus* only. Based on the results, we can conclude that the fruits demonstrate good antioxidant and antimicrobial potential.

## 1. Introduction

*Laurocerasus officinalis* Roem. is a species of flowering plant in the *Rosaceae* family and subfamily *Prunoideae*. It is also known by the synonyms, such as *Prunus laurocerasus* and laurel cherry.

The laurel cherry bush is an evergreen plant growing 2 to 6 m tall with ovoid dark purple to bright black fruits when ripe, 8–20 mm in diameter. The fruits are oval-round with a conical, elliptical, or round seed, similar to grape berries; 8–10 mm in diameter; and resemble cherries and ripen in clusters in the summer (July and August) [1,2,3].

The plant grows both naturally and under cultivation in the Black Sea region.

*L. officinalis* is an evergreen shrub, used for landscaping public open spaces—parks, gardens, and private properties [4,5,6].

The cyanogenic glycoside prulaurasin is found in the fruit, kernels, and leaves of *Laurocerasus officinalis* Roem. The compound’s distribution changes significantly as the fruit ripens; although its concentration in the kernels increases quickly during development, the initially high concentration in the pulp gradually decreases and is undetectable in fully ripe fruits, making the edible fruit safe [7].

The fruits of the cherry laurel are consumed fresh or dried; pickled; in juice, jam, or marmalade; and as beverages, or vinegar [8,9,10,11,12].

In Turkish alternative medicine, for example, the fruit is used to treat a number of diseases, such as sore throat; asthma; cough; stomach pain and ulcers; eczema; hemorrhoids; digestive and respiratory disorders; bronchitis; to strengthen bones; to expel kidney stones; to establish the acid-base balance of the blood; and also as a diuretic, antitussive, and antispasmodic agent [3,4,13,14,15,16,17,18,19,20]. The fruit extract demonstrates a good effect in the healing of open wounds. This effect was investigated in vivo and in vitro using an excisional wound model in mice [21].

Leaf ethanol and water extracts of *Laurocerasus officinalis* Roem have been found to have anti-inflammatory, antifungal, and anti-nociceptive effects [22,23].

The antimicrobial activity of cherry laurel was an essential part of our investigation. To the best of our knowledge, only a few reports have been published on the bioactive contents and antimicrobial activity of cherry laurel fruits [21,24,25].

Approximately 1.6 million people die each year from fungal infections, which is more than three times the projected number of deaths from malaria and comparable to tuberculosis [26]. Fungal infections have changed from being a rare cause of disease to a major contributor to human morbidity and mortality worldwide as a result of the HIV epidemic and medical advancements over the past few decades that have increased the number of immunocompromised people [27].

Human pathogenic fungi can cause invasive, cutaneous, and mucosal infections [28]. Compared to bacterial infections, invasive fungal infections are currently treated with fewer medications [29]. The rising mortality and morbidity caused by fungal infections are also linked to the limited number of currently available antifungal medications and antifungal resistance [30,31,32].

Therefore, the current study aims to investigate the antioxidant and antimicrobial potential of cherry laurel fruit extracts.

## 2. Materials and Methods

### 2.1. Plant Material

Cherry laurel fruits were collected in August 2024 from the Voivodinovo village, region of Plovdiv, Bulgaria (42°20′ N. 24°80′ E, GPS Coordinates). The harvested fresh fruits were directly used to prepare extracts. The plant material was provided by the Agricultural University—Plovdiv and was determined according to Delipavlov et al. and Stoyanov et al. [33,34]. All the solvents and chemicals were of analytical grade.

### 2.2. Fruit Extraction

For the extraction of phytochemical compounds, the fruits were extracted with 80% (*v*/*v*) aqueous methanol, 50% (*v*/*v*) aqueous ethanol, 70% (*v*/*v*) aqueous ethanol, 96% (*v*/*v*) aqueous ethanol, and water (hydromodul was 1:5). The extraction procedure was performed in an ultrasonic bath (VWR, Malaysia, VWR Singapore Pte Ltd., Singapore, 45 kHz and 30 W) at 45 °C for 15 min [35].

### 2.3. Geometric Parameters

The mass of the cherry laurel fruits was determined with a 0.001 g sensitivity of an electronic balance. The trilinear dimensions, namely major axis (L), mean axis (T), and minor axis (W), were measured by using a ruler with 0.1 mm sensitivity to determine the average size of the samples. The geometric mean diameter (GMD), sphericity (Φ), and aspect ratio (AR) were calculated using the following respective formulas [3,36,37].

GMD = (LWT)^1/3^, (1)

Spherisity = (GMD)/L.100,(2)

Surface area = π(GMD)^2^ (3)

### 2.4. Color Characteristics

The color of the laurel cherry fruits was determined with a colorimeter FRU WR-10QC (Anhui Haochuang Instrument Co., Anhui, China) and recorded in the L-a-b color system [38]. The color was determined by entering the data from L-a-b in a color available online (http://www.colormine.org/color-converter (accessed on 2 August 2025)). Chroma (C-ab) and hue (h°) were determined from the a and b values using Equations (4) and (5) [39].

C = sqrt (a*^2^ + b*^2^) (4)

h° = arctan (b/a)(5)

The total soluble solids (TSS) content was determined by a hand refractometer ATC (XDC Co., Ltd., Shanghai, China) and expressed as °Brix [40]. The pH values were assessed by a pH meter WTW pH 7110 (WTW, Weilheim in Oberbayern, Germany) equipped with a glass electrode, which was immersed directly into the laurel cherry fruit juice at 23 °C [41]. Titratable acidity (TA) was measured by titration of 2 mL with 0.1 M NaOH (Merck (Merck KGaA, Darmstadt, Germany) using phenolphthalein as an indicator (Merck (Merck KGaA, Darmstadt, Germany)) to the pH value of 8.1, and the result was expressed as g of malic acid equivalent per 100 g fresh weight (fw) [42]. Maturity index (MI) was measured as the TSS value divided by TA [43].

For the moisture content, the fruits were dried at 105 ± 1 °C to a constant weight [44].

For the determination of ash content, the pulverized samples were placed in a crucible, ignited in a muffle furnace at 550 °C to a constant weight. Then, it was cooled in a desiccator and weighed at room temperature to obtain the weight of the ash [45].

The polyuronide content and the degree of esterification of pectin were determined according to [46,47].

### 2.5. Celullose

The quantitative estimation of cellulose was performed gravimetrically [48].

Total lipid content (%) was determined by exhaustive Soxhlet extraction (AOAC methods) [49].

The total anthocyanin content (TAC) was estimated using the pH differential method [50].

### 2.6. Sugar and Polyol Content by HPLC-RID Method

The chromatographic separations and determination of sugars in the sample were performed on a high-performance liquid chromatograph, HPLC Elite Chrome Hitachi, equipped with a pump LC-20 AD, a column thermostat, a refractive index detector (RID) Chromaster 5450 (WVR, Hitachi, Tokyo, Japan), and software. The separation was carried out on the Shodex^®^ Sugar SP0810 (7 μm, 300 × 8.0 mm i.d., Tokyo, Japan) column with Pb^2+^ and a guard column Shodex SP-G (5 μm, 6 × 50 mm), operating at 80 °C, mobile phase H_2_O with a flow rate of 0.5 mL/min, and an injection volume of 20 μL. All the samples before injection were filtered through ISO Lab (ISOLAB Laborgeräte GmbH, Eschau, Germany) filters with a diameter of 4 mm and a pore size of 0.45 μm. The detection of sucrose, glucose, fructose, and sorbitol was performed in the linear range of 0.5–10 mg/mL, and the linearity of standard curves was determined (R^2^ > 0.998) [51].

### 2.7. Total Phenols and Flavonoids Content

The fruit extracts (0.2 mL) were mixed with 1 mL of five-fold diluted Folin–Ciocalteu reagent and 0.8 mL of 7.5% Na_2_CO_3_. After 20 min, the absorbance was measured at 765 nm against a blank sample. The results were expressed as mg equivalent of gallic acid (GAE) per g fresh and dry weight. The total flavonoid content was analyzed colorimetrically using Al(NO_3_)_3_ reagents [52]. The results were expressed as mg equivalents of quercetin (QE) per g dry and fresh weight.

### 2.8. Antioxidant Activity

DPPH radical scavenging ability: The fruit extracts (0.15 mL) were mixed with 2.85 mL fresh 0.1 mM methanol solution of DPPH. The samples were incubated at 37 °C in darkness for 15 min. The reduction in absorbance was measured at 517 nm against a blank, and % inhibition was calculated [52].

ABTS+ radical scavenging ability: The ABTS+ solution (2.85 mL) was mixed with 0.15 mL of the extracts. After 15 min at 37 °C in darkness, the absorbance was measured at 734 nm against ethanol [52].

FRAP assay: The FRAP reagent was prepared before analysis by mixing 10 parts 0.3 M acetate buffer (pH 3.6), 1 part 10 mM 2,4,6-tri(2-pyridyl)-s-triazine in 40 mM HCl, and 1 part 20 mM FeCl_3_ ·6 H_2_O in distilled water. The FRAP reagent (3.0 mL) was mixed with 0.1 mL of fruit extracts. After 10 min at 37 °C in darkness, the absorbance of the sample was measured at 593 nm [52].

### 2.9. Antimicrobial Activity

Tested microorganisms

Twenty microorganisms, including six Gram-positive bacteria (*Bacillus subtilis* ATCC 6633, *Bacillus amyloliquefaciens* 4BCL-YT, *Staphylococcus aureus* ATCC 25923, *Listeria monocytogenes* NBIMCC 8632, *Enterococcus faecalis* ATCC 19433, and *Micrococcus luteus* 2YC-YT), six Gram-negative bacteria (*Salmonella enteritidis* ATCC 13076, *Salmonella typhimurium*, *Klebsiella* sp.—clinical isolate, *Escherichia coli* ATCC 25922, *Proteus vulgaris* ATCC 6380, and *Pseudomonas aeruginosa* ATCC 9027), two yeasts (*Candida albicans* NBIMCC 74 and *Saccharomyces cerevisiae* ATCC 9763) and six fungi (*Aspergillus niger* ATCC 1015, *Aspergillus flavus*, *Penicillium* sp., *Rhizopus* spp.—plant isolates, and *Fusarium moniliforme* ATCC 38932) and *Mucor* from the collection of the Department of Microbiology at the University of Food Technologies, Plovdiv, Bulgaria, were selected for the antimicrobial activity test.

Luria–Bertani agar medium with glucose (LBG agar) was used for the cultivation of test bacteria. Malt extract agar (MEA) was used for the cultivation of test yeasts and fungi. The culture media were prepared according to the manufacturer’s instructions (Scharlab SL, Barcelona, Spain) and autoclaved at 121 °C for 20 min before use [53].

The antimicrobial activity of the extracts was determined by the agar well diffusion method [54]. After allowing the inoculated agar media to harden at room temperature, six wells (d = 6 mm) per Petri plate were made, and duplicates of 60 μL of the extracts were pipetted into the agar wells. The plates were incubated under identical conditions.

The antibiotics Penicillin (against bacteria) and Nystatin (against yeast and fungi), in the same concentration as the extracts, were used as controls.

### 2.10. Statistical Analysis

All the analyses were performed in triplicate (*n* = 3). The data were presented as mean values ± standard deviation (SD). Statistical analysis was performed using MS Excel 2010.

## 3. Results and Discussion

### 3.1. Physical Characteristics

#### 3.1.1. Geometric Parameters Measurements

Biometric studies are important for the determination of plant patterns in breeding programs. This is especially useful for varieties that are being introduced to new conditions. The cultivation of native varieties primarily relies on technical knowledge and specific varieties and environmental factors [3]. Biometric characterization can target the entire plant, or specific features like fruit, its stones, or seeds [3].

The geometric and volumetric characteristics of the Novita cherry fruit (15 fruits) are given in Table 1.

The length, width, and thickness of the fruit were 14.7 mm, 12.2 mm, and 15.1 mm, respectively, and differed by 1–2 mm from the measured parameters of the genotype 55 K 07 grown in Samsun, Turkey (13.1 mm, 13.9 mm, and 12.9 mm) [3]. However, they were significantly smaller than the parameters of the other two genotypes, 54 K 01 and 61 K 04, from the same region (17.4/19.6 mm (L), 17.7/17.0 mm (W), and 16.9/15.9 mm (T)) [3].

The geometric mean diameter (GDM), sphericity, and area (S) were 13.9 mm, 94.6% and 6.1 cm^2^, respectively. The GDM parameter is slightly higher than that of 55 K 07 (13.3 mm), but is significantly smaller than the measurements for fruits of genotypes 54 K 01 (17.2 mm) and 61 K 04 (17.4 mm). This parameter is also associated with the moisture content, which in the Novita variety is 74.2% and is higher than the value for genotype 55 K 07, 61.29% [3].

Sphericity indicates that the shape of the biological materials is spherical and thus facilitates rolling on the surface. The sphericity is close to 54 K 01 (99.17%) and 55 K 07 (99.99%), and the area to 55 K 07 (5.57 cm^2^) [3].

#### 3.1.2. Color Characteristics

The color of any food is a crucial attribute. The color coordinates of the cherry laurel fruit peel (L*, a*, b*) are shown in Table 2. A colorimeter was used to measure the brightness (L*), a* red color (+), and b* yellow color (+) parameters. Equations (4) and (5) were then used to determine the C* chromaticity (saturation) and h° color hue based on their values.

The brightness value (L*) for the fruit surface is 26.38 and is comparable to the value for the fruit of genotype 54 K 01, while for the fruits of genotype 55 K 07 and 61 K 04, the measured value is lower, 18.17 and 15.49, respectively. The a* and b* values of the three fruits from Samsun, Turkey, are significantly higher than those of the fruit of the Novita variety. The obtained color characteristics of the fruit skin were lower than the color parameters of the cherry laurel TRZ6 genotype grown in Trabzon in the Black Sea region in Turkey [4].

It has been found that there is a large difference in color parameters between different cherry laurel genotypes [3,4]. Based on the skin color measurements, the measured cherry laurel fruit variety Novita was black in color. The color of stone fruits such as plums and cherries is associated with the concentration of anthocyanins, which increases during ripening [55]. In different plum varieties, it has been observed that the total anthocyanin concentration is higher in the peel than in the pulp [55]. The measured amount of anthocyanins in the fruit of the cherry laurel, variety Novita, expressed as mg cyanidine-3-glycoside, is 12.84 mg/100 g fruit. Large variations in anthocyanin concentrations from 2 to 300 mg/100 g have been found in different sweet cherry varieties [55].

### 3.2. Chemical Composition

#### 3.2.1. Titratable Acidity (TA), pH, Total Soluble Solids (TSS), Maturity Index, Moisture, and Ash

The parameters TSS, TA, and the TSS/TA ratio are related to the quality of the fruit [56].

Titratable acidity (TA) is an indicator of how organic acids in fruits affect their flavor. The type of organic acids, such as malic, citric, tartaric, quinic, oxalic, fumaric, and succinic acids, each with a unique flavor, contributes to the overall flavor of the fruit [57]. Malic acid is the main organic acid in fruits belonging to the *Rosaceae* family, both in the genera *Prunus* (plum, apricot, peach, nectarine, and cherry) and *Malus* (apple and pear). For these fruits, the total acidity is very high at the beginning of development with an accumulation of malic acid during the first phase of rapid growth, but decreases during the ripening process, except for sweet cherry, which shows a continuous increase in acidity during ripening [58]. Furthermore, TA is a parameter that determines the quality of foods during their storage [59].

The total acidity of the fruits of cherry laurel (*Laurocerasus officinalis*) usually falls in the range of 0.12 to 0.70 g malic acid per 100 mL fruit juice (Table 3). This acidity is mainly due to the presence of malic acid, which is the dominant organic acid in the fruit [60].

In contrast to the fruits grown in the northwest of Turkey (Kocaeli, Sea of Marmara) [5], the TA calculated for the studied cherry laurel fruit is 0.45 ± 0.01 and is consistent with the TA calculated for the genotypes of cherry laurels in the northeastern Black Sea region [4]. The fruit’s measured pH value exhibits the same pattern. The pH level has a significant impact on the growth of microbes in a particular diet. The fruit of the Novita variety has a pH of 4.54 ± 0.01 (Table 3), which is the same as the fruit cultivated in the northeastern Black Sea region (pH 4.5) [14].

In stone fruits, sweetness is important and is measured using a refractometer and expressed as the concentration of soluble solids (sugars, salts, amino acids, proteins, acids, soluble substances, etc.) (TSS) in Brix units, where 1 Brix = 1 g sucrose equivalent per 100 g solution. In stone fruits, TSS increases during ripening and represents one of the largest characteristic biochemical changes in the fruit that occur before harvest. TSS is one of the most widely used indices of fruit ripeness in nectarines, peaches, apricots, and plums, and for these fruits, the TSS value is in the range of 5–25 Brix% [61].

The TSS content in cherry laurel genotypes in the Black Sea region of Turkey varies in the range of 8.6–21.3%. In our results, the TSS value of the Novita variety is 17.67 ± 0.58% (Table 3) and is rather close to the value of the genotypes grown in the northeastern part of the Black Sea region (Rize, Trabzon, Giresun, and Ordu) [62,63].

The parameters TSS, TA, and their ratio (TSS/TA) are strongly related to fruit quality. This ratio helps to assess the balance between sweetness (TSS) and tartness (TA) in fruits [64]. During the ripening process, the organic acids of stone fruits are degraded, the sugar content increases, and the sugar/acid ratio reaches a higher value. High acidity levels, often found in underripe fruits, make the fruit taste sour. A lower sugar/acid ratio in fruits indicates a sour taste, while a higher ratio indicates a sweet taste [65,66]. Overripe fruits have very low levels of fruit acid and therefore lack a characteristic taste. Sugars and organic acids in fruits and their products affect not only the taste but also the stability, perception, and maintenance of quality. In stone fruits, total soluble solids (TSS) mainly determine sweetness, while total acidity contributes to tartness and overall flavor. The TSS/acidity ratio is a key indicator of fruit quality and sensory ripeness, influencing the sweet/tart balance [48,67].

The calculated TSS/TA ratio for variety Novita was 39.27, and it is comparable to one of the fruit genotypes studied by Sulusoglu et al. [5] and Islam et al. [63].

The ash content varies depending on the variety and the region where the fruit is grown [68]. In the present study, the ash value (0.83%) of cherry laurel is close to that of “Kiraz” (0.75%), it is twice as high as the determined value of “Findik” (0.43%) and is more than three times higher than the ash content of the fruit from Akcaabat, Trabzon, Turkey (0.26%) [14]. The determined ash content is more than three times higher compared to the fruit collected from Akcaabat, Trabzon [14].

The moisture content of the examined fruits was higher compared to the cherry laurel genotypes, where the following moisture values were reported: 60.38, 61.29, and 64.13% [6], but also corresponds to the fruits from Akcaabat, Trabzon, Turkey [14].

#### 3.2.2. Polyuronide Content, Degree of Esterification of Pectin, Cellulose, and Total Lipids of Laurel Cherry, Variety Novita Fruit

Dietary supplements containing pectin may have decreased serum total cholesterol, decreased low-density lipoprotein cholesterol, and moderated the glucose response. Therefore, pectin and fibers are important food components due to their role in preventing colon diseases [69,70].

The determined polyuronide content (3.33 ± 0.23%) is in accordance with that determined in the fruits of the laurel cherry grown in Akcaabat, Trabzon, Turkey (3.2 ± 0.4%) [14]. Pectin was categorized as highly esterified [71] based on the degree of esterification that has been found (Table 4). This is significant for its gelling capabilities [72], which are essential when making jams and other sweets from the laurel cherry fruit.

The cellulose content in the studied fruits was 0.36 ± 0.08. The content of total lipids, on the other hand, was 0.85% (Table 4). This is significantly higher than that reported by Kalyoncu et al. (0.001%) [73].

#### 3.2.3. Sugar Content in the *Laurocerasus officinalis*, Novita Fruits

**Figure 1:**
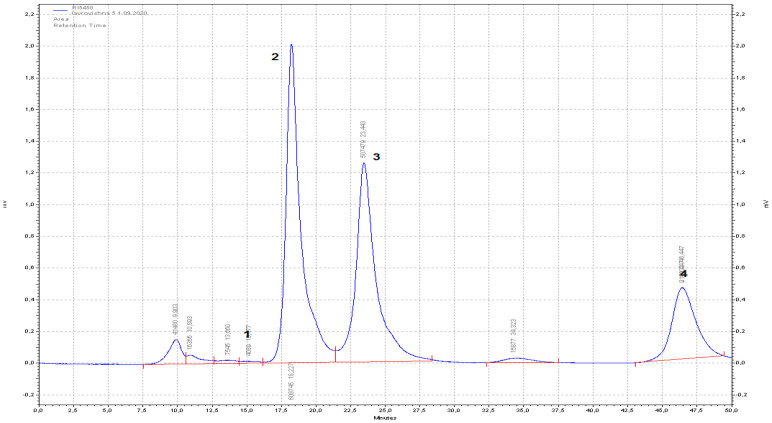
HPLC-RID chromatogram of cherry laurel water extract (1. sucrose, 2. glucose, 3. fructose, and 4. sorbitol).

The levels of sugars and organic acids are important factors in determining the taste of ripe fleshy fruits. In most fruits, sugar accumulation is an early event during their growth. The main accumulated sugar, depending on the type of fruit, is glucose, being the main one for grapes, and fructose for berries, cherries, mangoes, and citrus species, while sucrose is the main sugar found in stone fruits such as apricot, plum, nectarine, and peach [56]. In our experiments, the sugar content in fresh *Laurocerasus* fruits was analyzed by the HPLC-RID method. Two monosaccharides (glucose and fructose), one polyvalent alcohol (sorbitol), and one disaccharide (sucrose) were found in the fruit, Figure 1. The highest amount found was for glucose (2.45%) and fructose (1.49%), followed by sorbitol (0.70%), and the lowest was sucrose (0.02%). Borsani et al. [74] noted that sorbitol is consumed early in the ripening process, followed by partial degradation of sucrose, accompanied by an increase in fructose and glucose.

Generally, in these fruits, glucose is the dominant monosaccharide, followed by fructose, and sucrose is present in trace amounts, which is fully consistent with the literature data [75].

The content and ratio of carbohydrates were consistent with the results reported by Esringu et al. for cherry laurel (*Laurocerasus officinalis* L.) species grown in Turkey. The authors found that in cultivated fruits, the amount of measured carbohydrates was higher than in wild ones [48]. Ayaz et al., on the other hand, reported five times higher amounts of glucose, fructose, and sorbitol in cultivated (*Laurocerasus officinalis* Oxygemmis, Globigemmis, and Angustifolia) and wild species (*Laurocerasus officinalis* Roem.), but the authors did not detect sucrose [6,8].

#### 3.2.4. Total Phenolic Content and Flavonoids

The phenolic content, flavonoid concentration, and antioxidant potential of laurel cherry fruit have all been well investigated, particularly in the area of Turkey, where the plant is grown, and the fruits are used in traditional cooking.

Cherry laurel fruit is a good source of phenolics, together with considerable antioxidant activity [76]. The content of polyphenols and flavonoids in five fruit extracts (96% ethanol, 70% ethanol, 80% methanol, 50% ethanol, and water) of cherry laurel was investigated. The fruit of cherry laurel is a rich source of phenolics such as Chlorogenic acid, 4-Hydroxybenzoic acid, Ferulic acid, gallic acid, Protocatechuic acid, p-Coumaric acid, Vanillic acid, Rutin, and quercetin [77], which support its biological activity, especially its potential as a source of antioxidants [78].

**Figure 2:**
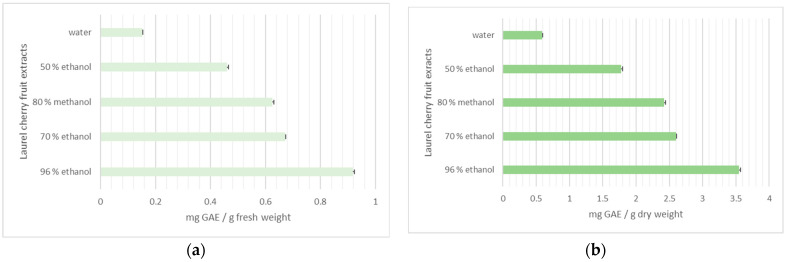
Total phenolic content (mg GAE/g) in laurel cherry extracts: (**a**) fresh weight, (**b**) dry weight.

Figure 2 presents the total polyphenol content (TPC) of the studied extracts. Since the fruit can be consumed both fresh and dried, the results for the total amount of flavonoids and polyphenols are shown for the fruit’s fresh and dry weight.

The content of bioactive components is presented for both fresh and dry weights. The 96% ethanol extract (0.91 mg GAE/g (fw)/3.53 mg GAE/g (dw)) had the highest content of polyphenols. The TPC decreased in the order of 70% ethanol (0.67 mg GAE/g (fw)/2.70 mg GAE/g (dw)), 80% methanol (0.62 mg GAE/g (fw)/2.40 mg GAE/g (dw)), 50% ethanol (0.46 mg GAE/g (fw)/1.76 mg GAE/g (dw)), and water (0.19 mg GAE/g (fw)/0.59 mg GAE/g (dw)).

**Figure 3:**
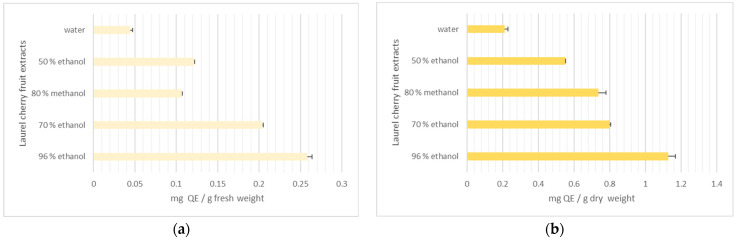
Total flavonoids (mg QE/g) in laurel cherry extracts: (**a**) fresh weight, (**b**) dry weight.

Figure 3 presents the flavonoid content of the studied extracts. The results are presented as a comparison of fresh and dry weight.

The amount of total flavonoid content in different extracts exhibits a similar tendency. Most of the flavonoids were present in the 96% ethanol extract (0.29 mg QE/g (fw)/1.13 mg QE/g (dw)). The lowest level of TFC was detected in the water extract (0.05 mg QE/g (fw)/0.21 mg QE/g (dw)).

Koc et al. reported a polyphenol content of 16.84 to 23.32 mg GAE/100 g dry weight in *Laurocerasus officinalis* fruits grown in the Eastern Black Sea region of Turkey [79]. This is about ten times lower compared to the TPF content, measured in our experiments for the Novita variety. Compared to the methanolic extract of cherry laurel fruit grown in the “Zelenicje” Nature Reserve, Serbian (42.2 mg GAE/g dry extract and 13.5 mg QE/g dry extract) [80], the 80% methanolic extract had a twenty-fold lower level of TPC and TFC at 2.40 mg GAE/g (dw) and 0.74 mg QE/g (dw). The content of polyphenols and flavonoids in the laurel cherry variety Novita is about ten times higher than in wild varieties grown in Turkey [79], but lower than in varieties grown in Serbia [80]. The reason for this is most likely that the variety is cultivated for decorative purposes and mostly for landscaping (hedges).

In comparison to our experiments, methanol fruit extracts from twelve genotypes of cherry laurel cultivated in the Sakarya region, Turkey, contained five to twenty-eight times more polyphenols [24]. Methanol extracts of cherry laurel fruits gathered in different regions of Turkey, Istanbul [81], Giresun province [18], and the Eastern Black Sea area [15] had three to ten times higher concentrations of polyphenols than the methanol extract isolated in our study. The aqueous extract of the fruit of the laurel cherry, variety Novita, contains ten times less phenols compared to the collected fruit from Düzce, Turkey [82].

Our results were in agreement with the previously published data regarding peach, nectarine, and plum grown in the Plovdiv region, which belong to the *Rosaceae* family, Genus *Prunus*. The TPC values for 80% methanol extracts of the peach varieties “Gergana,” “Ufo4”, “July Lady,” and “Laskava” were 0.43 mg GAE/g, 0.34 mg GAE/g, 0.58 mg GAE/g, and 0.58 mg GAE/g [83]. Marinova et al. also reported a polyphenol content in peach (50.09 mg GAE/100 g) that was consistent with our results [84]. Our results also coincide with reports for total phenols for plum (*Prunus domestica*) (64.5 mg GAE/100 g) [70,85]. TPC levels in Chinese peaches and nectarines ranged from 340.1 mg GAE/kg to 820.3 mg GAE/kg, which were comparable to our results [86]. Additionally, a number of elements, including genotype, agronomic techniques, harvest ripeness, postharvest storage, meteorological conditions, and geographic regions, affect the overall phenolic content of the plant [79].

### 3.3. Biological Experiments

#### 3.3.1. Antioxidant Activity Measurements

Three different techniques, DPPH, ABTS, and FRAP, were used to evaluate the antioxidant activity of the fruit extracts. DPPH and FRAP use single-electron transfer to determine antioxidant activity, whereas ABTS combines single-electron transfer with hydrogen atom transfer.

The results are presented in Table 5. The highest radical scavenging activity by the DPPH method was shown by the 96% ethanol extract, followed by the 70% ethanol, 80% methanol, 50% ethanol, and the water fruit extract. This trend is observed with the other two methods, ABTS and FRAP. Since the fruit can be consumed both fresh and dried, the results for the total amount of flavonoids, polyphenols, and antioxidant activity are shown for the fruit’s fresh (^a^) and dry (^b^) weight.

In the fruit extracts, the highest antioxidant values corresponded to the highest phenolic and flavonoid concentrations. Our results regarding the DPPH and ABTS analysis of the methanol extracts, and the DPPH analysis of the water extracts, are in accordance with the data reported by Celik et al. [76], while for the aqueous extracts, we report ten times lower results measured by the ABTS method.

The antioxidant activity of 80% methanolic extracts, presented by the ABTS and FRAP methods, demonstrates ten times higher activity compared to that reported by Capanoglu et al. [87].

The results of the antioxidant activity of 70% ethanolic extracts of laurel cherry are consistent with apples belonging to the *Rosaceae* family, variety Florina, grown in orchards from Plovdiv and Brestnik village −12.07 mM TE/g dw measured by DPPH method; 33.24 mM TE/g dw measured by ABTS method; and 19.63 mM TE/g dw measured by FRAP method [78].

The content of polyphenols and the demonstrated antioxidant activity in the fruits of the ornamental shrub *Prunus laurocerasus* are in full agreement with some fruits of the *Rosaceae* family that are present in the daily diet. Therefore, the fruits could be a source of biologically active substances with antioxidant potential.

#### 3.3.2. Correlation Between the Total Phenolic Content, Total Flavonoid Content, and the Antioxidant Activity

The antioxidant capacity is most strongly influenced by the total phenolics and total flavonoid concentrations. A statistical indicator of the strength of a linear link between two variables is the correlation coefficient. The correlation showed the relationship between polyphenols (TPC and TFC), polyphenols, and antioxidant methods, and between the antioxidant methods themselves. The correlation between TPC, TFC, and antioxidant activity of the studied extracts measured by the three methods (DPPH, ABTS, and FRAP) is presented in Table 5. It was established that the radical scavenging activity of the studied extracts is highly correlated with the content of total phenols and flavonoids. TPC and TFC contribute to the antioxidant properties of the tested fruit extracts [88]. A positive linear correlation (r^2^) between the results obtained by the different antioxidant methods was found.

A similar observation for the positive dependence of the antioxidant potential, investigated by total phenolic content, was reported by Petkova et al. for Florina apple (coefficient of correlation r^2^ = 0.9801, 0.9462, and 0.9994 for ABTS, DPPH, and FRAP values, respectively) [53,89].

#### 3.3.3. Antimicrobial Activity Results

Antibiotic resistance is currently one of the world’s most persistent issues, and several powerful drugs have failed to control diseases. Aggregated bacteria that adhere to surfaces and are covered in an extracellular polymeric matrix are called biofilms. Within a biofilm, the local environment shields persister cells from the immune system [90]. These bacterial biofilms can form on any surface, including medical devices and hospital water distribution systems [91]. Therefore, the current study aimed to evaluate the antimicrobial and antifungal activity of laurel cherry fruit extracts.

There is rather little information in the literature regarding the antibacterial activity of the fruit against a limited number of microorganisms. Özçelik et al. reported antibacterial activity of alcoholic fruit extracts only against four strains of Gram-positive bacteria, while we observed an inhibitory effect of fruit methanolic extracts against tested Gram-positive, Gram-negative bacteria, yeasts, and fungi [92].

The antimicrobial activity of the aqueous and methanolic extracts of the fruits of the laurel cherry was studied against six Gram-positive and six Gram-negative bacteria. In our experiments, the aqueous extracts did not exhibit antimicrobial activity against *B. subtilis*, *B. amyloliquefaciens*, *S. aureus*, *L. monocytogenes*, and *E. faecalis*, while the methanolic extracts showed moderate sensitivity (Table 6).

The aqueous extracts were moderately sensitive, and the methanolic extracts were highly sensitive against *M. luteus*. Concerning the Gram-negative bacterial strains, the methanolic extracts were moderately sensitive against five of them—*S. enteritidis*, *S. typhimurium*, *E. coli*, *P. vulgaris*, and *P. aeruginosa*—and were highly sensitive against *Klebsiella* sp. The methanolic extracts inhibited the growth of five of the tested fungi—*A. niger*, *A. flavus*, *Penicillium* sp., *Rhizopus* spp., and *F. moniliforme*—and showed high sensitivity against them (Table 6).

The fact that the quantity of polyphenolic components predominates in the methanol extract while sugars predominate in the aqueous solutions is a plausible reason for the methanol extract’s superior antibacterial action.

**Figure 4:**
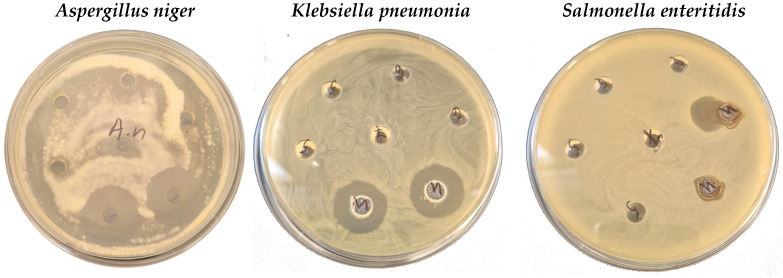
Selected petri dish photos of antimicrobial and antifungal activity assay of cherry laurel extracts.

The methanol extracts showed strong sensitivity to five of the tested fungi—*A. niger*, *A. flavus*, *Penicillium* sp., *Rhizopus* spp., and *F. moniliforme*—and did not inhibit the growth of *C. albicans* and *Mucor* sp. The aqueous extracts did not show activity against the tested fungi and yeast *S. cerevisiae*. The methanol extracts showed moderate activity against yeast (Figure 4). Methanol, used as a control, did not show any antimicrobial effect.

In contrast to our research, Celik et al. found that aqueous and methanolic extracts of lyophilized cherry laurel powder did not exhibit antibacterial activity against *Staphylococcus aureus* NCTC 8530, *Listeria monocytogenes* ATCC 7644, and *Escherichia coli* BL21 [76]. Ayla et al. reported strong antibacterial activity of cherry laurel fruit extracts against both Gram-positive bacteria (*Bacillus cereus* and *Staphylococcus aureus*) and Gram-negative bacteria (*Escherichia coli*, *Klebsiella pneumonia*, and *Pseudomonas aeruginosa*) [21].

## 4. Conclusions

The present study investigates the phytochemical composition and biological activities of the fruits of the Novita shrub, grown as an ornamental plant in the South of Bulgaria. The study is significant since it examines the phytochemical profile of this variety’s fruits for the first time.

The fruits can be consumed as a novel food ingredient containing dietary fiber (cellulose and pectins), sugars (glucose, fructose, and sucrose), natural pigments (anthocyanins), and phenolic compounds. The results of this study showed that the fruits contain low levels of sucrose, dietary fiber, and moderate total phenolic content. Based on its antioxidant and antimicrobial potential, laurel cherry extracts can be incorporated into dietary supplements. For a better understanding of its potential as an antimicrobial agent, further research on the synergy between *Prunus laurocerasus* and traditional antibiotics is necessary.

## Figures and Tables

**Table 1:** Weight and geometrical parameters of laurel cherry fruit.

Parameters	
Weight (g)	1.6 ± 0.2
Width (mm), W	12.2 ± 0.2
Length (mm), L	14.7 ± 0.3
Thickness, (mm) T	15.1 ± 0.4
GDM, (mm)	13.9 ± 0.1
Spherisity, (%)	94.6 ± 2.2
Surface area, (cm^2^)	6.1 ± 0.1

**Table 2:** Color parameters.

Color Parameters	Values
L*	26.38 ± 0.28
a*	0.89 ± 0.02
b*	0.11 ± 0.01
C*	0.91 ± 0.07
hue angle h°	13.09 ± 0.64

**Table 3:** Values of Titratable acidity (TA), pH, TSS, moisture, and ash in the fruits of the laurel cherry, variety Novita.

Parameter	Value
pH	4.54 ± 0.01
total acidity (%)	0.45 ± 0.01
TSS, Brix (%)	17.67 ± 0.58
moisture (%)	74.19 ± 1.19
ash (%)	0.83 ± 0.09

**Table 4:** Polyuronide, degree of esterification of pectin, cellulose, and total lipid content.

Parameter	Value
polyuronic acid content (%)	3.33 ± 0.23
degree of esterification (%)	56.47 ± 7.01
celulose (%)	0.36 ± 0.08
total lipids (%)	0.85 ± 0.04

**Table 5:** Antioxidant activity (mM TE/g) of the tested laurel cherry extracts.

	DPPH	ABTS	FRAP
96% ethanol	5.90 ^a^ ± 0.54	8.28 ^a^ ± 0.16	6.79 ^a^ ± 0.23
	22.85 ^b^ ± 0.83	32.04 ^b^ ± 0.25	26.28 ^b^ ± 0.91
70% ethanol	4.46 ^a^ ± 0.22	6.27 ^a^ ± 0.30	5.06 ^a^ ± 0.04
	17.29 ^b^ ± 0.73	24.27 ^b^ ± 0.14	19.59 ^b^ ± 0.16
80% methanol	4.31 ^a^ ± 0.80	5.60 ^a^ ± 0.40	3.77 ^a^ ± 0.05
	16.70 ^b^ ± 0.91	21.69 ^b^ ± 0.18	14.60 ^b^ ± 0.18
50% ethanol	2.78 ^a^ ± 0.23	4.14 ^a^ ± 0.14	2.83 ^a^ ± 0.14
	10.76 ^b^ ± 0.67	16.04 ^b^ ± 0.54	10.95 ^b^ ± 0.67
water	0.45 ^a^ ± 0.05	0.97 ^a^ ± 0.02	0.57 ^a^ ± 0.01
	1.76 ^b^ ± 0.07	3.75 ^b^ ± 0.34	2.24 ^b^ ± 0.19

The results for the antioxidant activity measured by the three methods are presented for fresh (^a^) and dry (^b^) weight of the fruit.

**Table 6:** Antimicrobial activity of water and methanol fruit extracts.

	Inhibition Zone, mm			
Tested Microorganism	Water Extract	Methanol Extract	Nystatin	Penicillin
*B. subtilis*	0 ± 0.00	13 ± 0.00	n.a.	-
*B. amyloliquefaciens*	0 ± 0.00	12 ± 0.00	n.a.	-
*S. aureus*	0 ± 0.00	17 ± 0.00	n.a.	30
*L. monocytogenes*	8 ± 0.00	15 ± 0.00	n.a.	20
*E. faecalis*	0 ± 0.00	13 ± 0.00	n.a.	-
*M. luteus*	15 ± 0.00	20 ± 0.00	n.a.	-
*S. enteritidis*	8 ± 0.00	17 ± 0.00	n.a.	-
*S. typhimurium*	0 ± 0.00	17 ± 0.00	n.a.	-
*Klebsiella* sp.	0 ± 0.00	20 ± 0.00	n.a.	-
*E. coli*	0 ± 0.00	13 ± 0.00	n.a.	-
*P. vulgaris*	0 ± 0.00	12 ± 0.00	n.a.	-
*P. aeruginosa*	0 ± 0.00	13 ± 0.00	n.a.	-
*C. albicans*	0 ± 0.00	0 ± 0.00	21	n.a.
*S. cerevisiae*	0 ± 0.00	12 ± 0.00	18	n.a.
*A. niger*	0 ± 0.00	25 ± 0.00	18	n.a.
*A. flavus*	0 ± 0.00	20 ± 0.00	18	n.a.
*Penicillium* sp.	0 ± 0.00	22 ± 0.00	n.a.	n.a.
*Rhizopus* spp.	0 ± 0.00	20 ± 0.00	n.a.	n.a.
*F. moniliforme*	0 ± 0.00	20 ± 0.00	15	n.a.
*Mucor* spp.	0 ± 0.00	8 ± 0.00	n.a.	n.a.

## Data Availability

The original contributions presented in this study are included in the article. Further inquiries can be directed to the corresponding authors.
